# MYB17‐DFR/LDOX Module Positively Regulates Cyanidin Deposition in *Cinnamomum Camphora*

**DOI:** 10.1111/pce.70664

**Published:** 2026-06-12

**Authors:** Shupei Rao, Xue Gong, Le Shen, Huihu Li, Bo Zhang, Yongda Zhong

**Affiliations:** ^1^ Jiangxi Provincial Key Laboratory of Improved Variety Breeding and Efficient Utilization of Native Tree Species, Institute of Resources and Environment Jiangxi Academy of Sciences Nanchang Jiangxi China; ^2^ Department of Forest Genetics and Plant Physiology, Umeå Plant Science Centre Swedish University of Agricultural Sciences Umeå Sweden

**Keywords:** anthocyanin, *Cinnamomum camphora*, metabolome, molecular mechanism, MYB

## Abstract

*Cinnamomum camphora* is a cornerstone ornamental tree species in southern China, valued for its evergreen foliage and distinctive scent. However, the uniformly green foliage and brown bark limit its landscaping values. In this study, we characterised a coloured camphor variety, ‘Gantong 1’, and revealed cyanidin content as the critical factor responsible for its stem coloration. Correlation analysis indicated that the transcription factor CcMYB17 plays a crucial role in cyanidin accumulation. We functionally validated *CcMYB17* through yeast one‐hybrid assays, luciferase reporter assays and multi‐omics analysis in a poplar model system. Our results demonstrate that CcMYB17 regulates the synergistic accumulation of cyanidin and delphinidin by activating key anthocyanin biosynthesis genes, specifically *CcDFR* and *CcLDOX*. This systematic elucidation of the colour mechanism ‘Gantong 1’ and the functional role of CcMYB17 deepens our understanding of ornamental trait improvement in *C. camphora*. It provides a foundation for molecular‐assisted breeding of coloured woody trees.

## Summary statement

This research explained the pattern of colour variation and revealed *CcMYB17‐CcDFR/CcLDOX* regulates the synergistic accumulation of cyanidin in a coloured camphor variety ‘Gantong 1’. It provides a foundation for molecular‐assisted breeding of coloured woody trees.

## 1 Introduction

*Cinnamonum camphora*, a broad‐leaved evergreen tree within the Lauraceae family, possesses significant ornamental, medicinal, and economic value. Renowned for its substantial carbon sequestration capability, this species is extensively utilised in road greening and landscape design, making it a prominent feature in southern China (Li et al. 2023). However, the most cultivated varieties are uniformly with green foliage and yellowish‐brown or grayish‐brown stem, which limits their appeal and economic potential as premium ornamental trees. Consequently, breeding new varieties with distinctive stem and leaf coloration is a major objective for enhancing the market value of *C. camphora*.

A previously identified natural mutant ‘Gantong 1’ (GT), exhibits bright red stems and branches throughout spring, winter, and early summer, with new leaves emerging orange (Zhong et al. 2022). These stable seasonal colour changes establish GT as an exceptional ornamental specimen with high commercial appeal. Furthermore, it serves as an ideal subject for investigating the molecular mechanisms of pigmentation. Studying this variety will advance our understanding of anthocyanin accumulation in camphor trees and provide essential genetic resources and knowledge for breeders. Ultimately, this research facilitates the development of targeted, high‐end ornamental varieties, thereby broadening the market for specialised *C. camphora* cultivars.

Anthocyanins are major secondary metabolites derived from the phenylpropanoid pathway and constitute the primary pigments responsible for the red, orange, purple, and blue coloration in plant organs (Yamagishi 2013; Moustaka et al. 2020; Xie et al. 2020; Wang et al. 2023). In nature, the predominant anthocyanin glycosides are derived from six core anthocyanin aglycones: pelargonidin, cyanidin, delphinidin, peonidin, malvidin, and petunidin (Anum et al. 2024). These pigments are synthesised through a specific branch of the flavonoid pathway.

The anthocyanin biosynthesis pathway, a well‐characterised segment of the flavonoid metabolism, is activated by a suite of anthocyanin biosynthesis genes (ABGs) (Liu et al. 2021; Sunil and Shetty 2022). These are categorised into early biosynthesis genes (EBGs) and late biosynthesis genes (LBGs). EBGs, such as *CHS, CHI, F3'H, FLS* and *F3H* promote the production of flavonols and anthocyanin precursors (Zhang et al. 2025); LBGs, including *DFR, ANS/LDOX, UGT, GT, RT, MT* and *AT* are responsible for the crucial later steps of anthocyanin biosynthesis and modification (He et al. 2023; Wang et al. 2023). The pathway diverges into three branches driven by *F3'H* and *F3’5'H*, ultimately yielding orange pelargonidin, red cyanidin, and blue delphinidin. Consequently, *F3'H*, *F3’5'H* and *DFR* are considered key determinants of colour variation in plants.

The anthocyanin biosynthesis pathway is primarily regulated by the MBW ternary complex, composed of MYB, bHLH and WD40 proteins. Among these, MYB transcription factors play a central role by binding directly to the promoters of anthocyanin structural genes (Wang et al. 2019; Sun et al. 2020; Yang et al. 2021; Li et al. 2022; Liu et al. 2022; Qin et al. 2022; Yang et al. 2022; Jiang et al. 2023). Key regulators, such as *AtMYB75/PAP1, AtMYB90/PAP2, AtMYB113, AtMYB114* in Arabidopsis, have been characterised, and their homologs have been shown to enhance anthocyanin accumulation in diverse plant species (Maier et al. 2013; He et al. 2023). Here, we show that CcMYB17, an AtMYB113 homolog, regulates anthocyanin accumulation and stem coloration in camphor tree by directly activating the key biosynthetic genes *CcDFR* and *CcLDOX*, revealing a central role for the CcMYB17‐DFR/LDOX module in this process.

## 2 Results

### 2.1 Dynamics of Chlorophyll, Carotenoids and Anthocyanins in Camphor Trees

**Figure 1:**
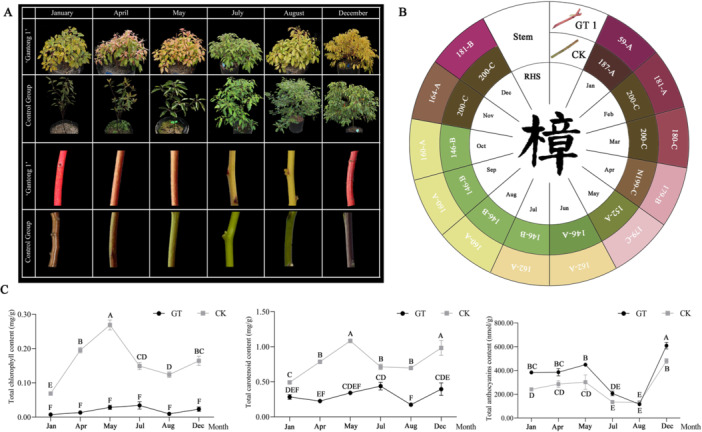
Seasonal coloration of GT and CK trees. (A) Representative pictures of seasonal colour changes of both GT and the control plants (CK). (B) RHS colour chart reflects the seasonal stem colour change. (C) Quantification of seasonal stem changes of pigment content. The data points represent mean values from duplicate studies with the error bars representing standard error, and were calculated from at least three repeat experiments. Different letters indicate significant differences (*p* < 0.05) by Duncan's multiple range test, comparing all groups including both genotypes across all seasons.

To investigate the seasonal coloration of the ‘Gantong 1’(GT) mutant, we evaluated its stem colour using the Royal Horticultural Society (RHS) colour chart (Figure 1A,B). A pool of nine half‐sib progenies from the same mother tree as the GT served as control plants (CK). The stem of GT trees exhibited a bright red hue (59‐A, 181‐A) from late October to early June, which gradually transitioned to an orange‐yellow colour (162‐A, 160‐A) during summer and early autumn (early June to early October) (Figure 1A,B). In contrast, the stem of CK trees was primarily dark brown (187‐A) and brown (200‐C) in winter, spring, and autumn, shifting to dark green (152‐A) or green (146‐A) in summer (Figure 1A,B). These observations confirm that GT stem is predominantly red, while CK is mainly green, revealing a significant phenotypic difference.

Chlorophyll, carotenoid, and anthocyanin are the primary pigments determining plant coloration. To elucidate the cause of the colour variation in GT and CK stem across seasons, we quantified their total content (Figure 1C). The results demonstrated significant seasonal fluctuations in the total content of chlorophyll, carotenoid, and anthocyanins in the stems of both GT and CK trees. While the trends for chlorophyll, carotenoids, and anthocyanins were consistent across stages in both lines, GT plants displayed significantly lower levels of chlorophyll and carotenoid compared to CK (Figure 1C). Conversely, anthocyanin content was significantly higher in GT, with prominent peaks in January, April, May, and December, and the highest levels were observed in May and December (Figure 1C). These results suggested that the content of anthocyanins is the primary factor, combined with the reduced chlorophyll and carotenoid levels determines the final colour of camphor tree stem.

### 2.2 Identification and Comparison of Anthocyanin Compounds

**Figure 2:**
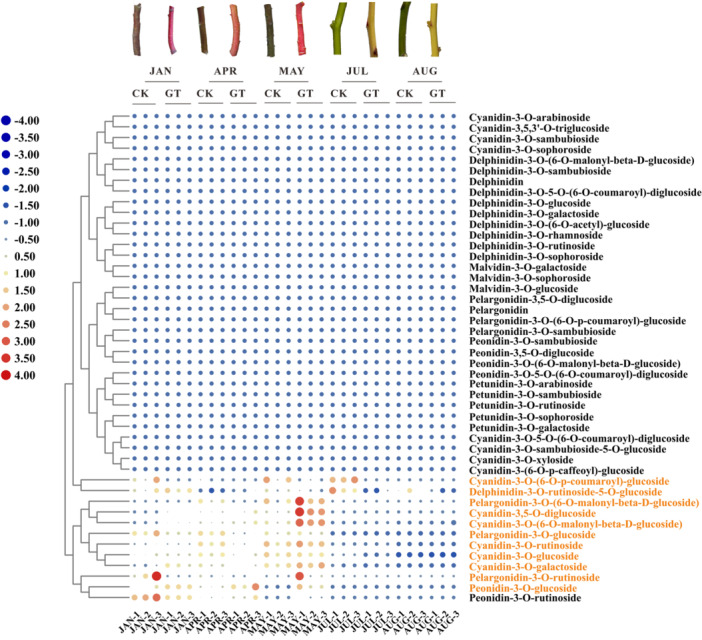
Content of anthocyanin targeted metabolites in GT and CK trees. The Dot plots on the left reflects the relative content trend changes of various anthocyanin metabolites. The size of dots represents the proportion of the anthocyanin targeted metabolites content, and the spectrum of colour indicates the mean levels (log_2_ transformed). The table in the middle shows the relative content of various anthocyanin targeted metabolites. The yellow box represents the main metabolites among all anthocyanin targeted metabolites (relative content > 0.5). [Color figure can be viewed at wileyonlinelibrary.com]

To identify the specific pigments responsible for coloration in the GT mutant, we performed anthocyanin‐targeted metabolomic analysis on year‐round stem samples from both GT and CK trees. A total of 46 anthocyanin metabolites were identified and categorised into eight classes. Among these, cyanidin‐based anthocyanins were the most abundant colouring substance in the stem. Metabolite clustering showed that Cyanidin‐3‐O‐glucoside and Cyanidin‐3‐O‐rutinoside has the highest content in camphor tree stem. Using a relative content threshold of > 0.5, 11 anthocyanin metabolites including Cyanidin‐3‐O‐glucoside, Cyanidin‐3‐O‐rutinoside, Cyanidin‐3‐O‐(6‐O‐malonyl‐beta‐d‐glucoside), Cyanidin‐3,5‐O‐diglucoside, Cyanidin‐3‐O‐galactoside, Cyanidin‐3‐O‐(6‐O‐p‐coumaroyl)‐glucoside, Delphinidin‐3‐O‐rutinoside‐5‐O‐glucoside, Pelargonidin‐3‐O‐(6‐O‐malonyl‐beta‐d‐glucoside), Pelargonidin‐3‐O‐glucoside, Pelargonidin‐3‐O‐rutinoside, Peonidin‐3‐O‐glucoside, as the main colouring metabolite in camphor trees stem (Figure 2, Supporting Information S13: Table S2).

**Figure 3:**
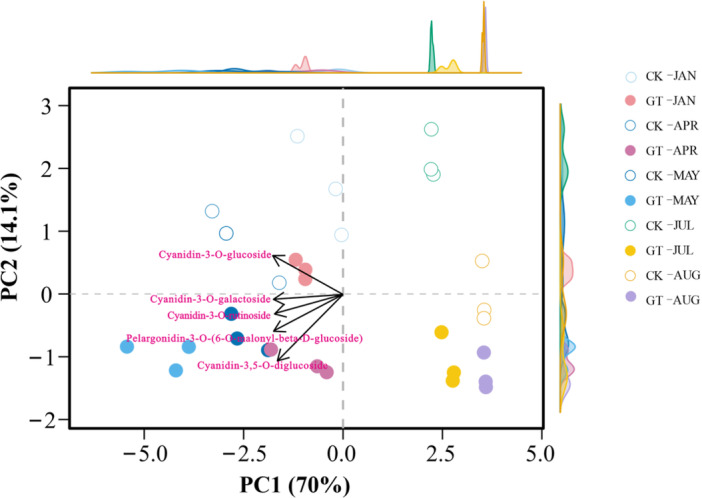
PCA analysis on the content of 11 core metabolites in GT and CK trees. The arrow represents the direction of the principal component, and the length of the arrow reflects the contribution of the principal component to the total variance of the data. The longer the arrow, the more central the component. [Color figure can be viewed at wileyonlinelibrary.com]

To determine which core anthocyanin affects the colouring effect of GT in stem, we conducted PCA analysis on the content of these 11 metabolites in all samples. The results showed that the cumulative contribution rate of the first principal component (PC1) reached 70% (Figure 3). Based on the load value, Cyanidin‐3‐O‐glucoside, Cyanidin‐3‐O‐galactoside, Cyanidin‐3‐O‐rutinoside, Pelargonidin‐3‐O‐(6‐O‐malonyl‐beta‐d‐glucoside), Cyanidin‐3,5‐O‐diglucoside were selected, indicating that these substances are of great importance in influencing GT coloration (Figure 3).

### 2.3 *CcMYB17* Promotes the Anthocyanin Accumulation

To investigate transcriptomic profiles underlying GT coloration, we performed RNA‐seq analysis on seasonal stem samples from both GT and control (CK) trees. KEGG enrichment analysis revealed that the differentially expressed genes (DEGs) were significantly enriched in the flavonoid biosynthesis pathway (Supporting Information S1: Figure S1). We identified 26 DEGs encoding 13 key enzymes involved in this pathway, including *CYP73A, HCT, CYP98A, CHS, CHI, CYP75B, F3H, F3'H, FLS, LAR, ANR, DFR* and *3‐OMT*, suggesting that the anthocyanin pathway is activated (Supporting Information S2: Figure S2).

**Figure 4:**
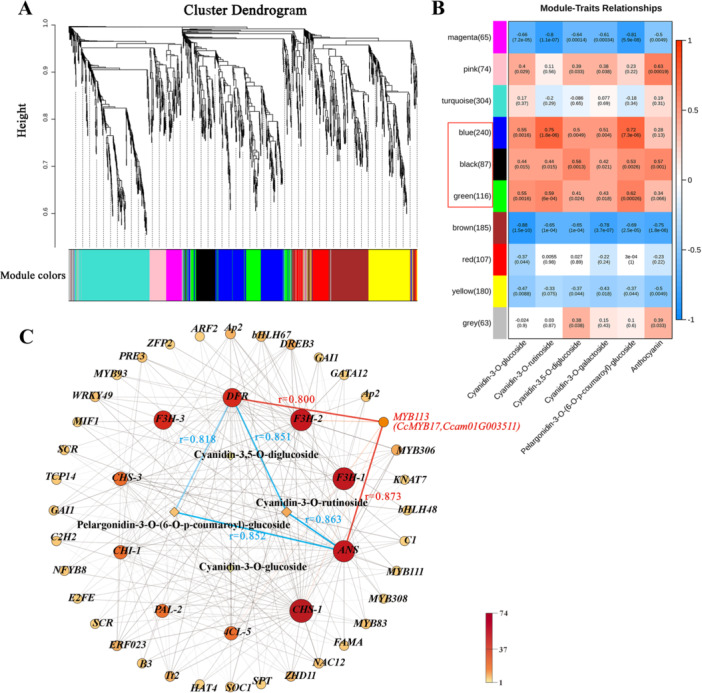
Co‐expression network analysis identifies modules and a key transcription factor associated with cyanidin accumulation. (A) Cluster dendrogram of genes from the GT and CK transcriptome, with assigned module colours indicated by the horizontal bars. (B) Module‐trait relationships showing the correlation coefficients and *p*‐values between each module's eigengene and the key anthocyanin metabolites: Cyanidin‐3‐O‐(6‐O‐malonyl‐β‐d‐glucoside), Cyanidin‐3‐O‐rutinoside, Cyanidin‐3‐O‐galactoside and total anthocyanin content. Modules with *r* > 0.5 and *p* < 0.01 were considered significant. The blue, black and green modules were significantly correlated with the metabolites. (C) A regulatory network illustrating the correlation between the candidate transcription factors, core anthocyanin structural genes, and the cyanidin derivatives. Node size is proportional to ktotal (intramodular total connectivity). CcMYB17 represents the transcription factor with the highest ktotal among all TFs in the three selected modules. [Color figure can be viewed at wileyonlinelibrary.com]

To identify the transcriptional regulators of anthocyanin accumulation in GT, weighted gene co‐expression network analysis (WGCNA) was performed based on transcriptome data and the contents of five critical anthocyanin metabolites (Cyanidin‐3‐O‐glucoside, Cyanidin‐3‐O‐galactoside, Cyanidin‐3‐O‐rutinoside, Pelargonidin‐3‐O‐(6‐O‐malonyl‐beta‐d‐glucoside), Cyanidin‐3,5‐O‐diglucoside) as well as total anthocyanins. Modules were defined based on a positive correlation coefficient threshold of *r* > 0.5 and a *p*‐value < 0.01 with at least one of the five key anthocyanin metabolites or total anthocyanin content (Figure 4B). Using these criteria, three co‐expression modules (blue, black, green) that were significantly correlated with the levels of these compounds and total anthocyanins (Figure 4A,B).

To identify the most central transcription factors governing anthocyanin accumulation, we calculated the intramodular total connectivity (ktotal) for each gene within the three selected modules (blue, black, green). ktotal measures the sum of correlation strengths between a given gene and all other genes in the same module, with higher values indicating greater network centrality. Among all transcription factors present in these modules, CcMYB17 exhibited the highest ktotal value (68.68), indicating its role as a hub gene within the co‐expression network (Supporting Information S15: Table S4). Consistently, CcMYB17 showed a strong Pearson correlation (*r* = 0.816) with the key anthocyanin metabolite Pelargonidin‐3‐O‐(6‐O‐p‐coumaroyl)‐glucoside. Moreover, the expression of *CcMYB17* was highly correlated with multiple key anthocyanin biosynthetic genes, including *4CL*, *CHS*, *DFR*, *LDOX* and *F3H* (*r* > 0.8, Figure 4C, Supporting Information S16: Table S5). As an ortholog of Arabidopsis anthocyanin regulatory factor, *AtMYB113*, this result suggested that *CcMYB17* serves as a core transcription factor regulating anthocyanin synthesis in camphor tree. Moreover, the key structural genes of anthocyanin biosynthesis, *DFR* and *LDOX*, showed high correlations with the major substance Cyanidin‐3‐O‐rutinoside (*r* = 0.851 and 0.863, Supporting Information S17: Table S6). In addition, the expression correlations between *CcMYB17* and *DFR* or *LDOX* were also significantly high (0.800 and 0.873). To validate the transcriptomic results, we performed qRT‑PCR analysis on *CcMYB17* expression in stems of GT and CK across all points of transcriptomes. qRT‐PCR profiles closely mirrored the RNA‐seq data, with *CcMYB17* transcript levels consistently and significantly higher in GT than CK in January, April and May (Supporting Information S3: Figure S3). This concordance between transcriptome and qRT‐PCR data, together with the strong correlation between *CcMYB17* expression and anthocyanin content, reinforces the central role of *CcMYB17* in regulating seasonal stem pigmentation. Altogether, these data suggested that CcMYB17‐DFR/LDOX is a pivotal module regulating coloration in camphor tree (Figure 4C).

Phylogenetic trees of MYB gene family of *Populus trichocarpa*, *Eucalyptus grandis* and *Arabidopsis thaliana* were constructed using iq‐tree 2.3.6. They are divided into six groups according to Pfam domain. CcMYB17, containing a typical R2R3‐MYB domain (PF00249), clusters closely with known anthocyanin‐promoting transcription factors from Arabidopsis (AtMYB75, AtMYB90, AtMYB113), poplar (PtrMYB113), and eucalypt (EgMYB1) (Supporting Information S4 and S5: Figures S4 and S5). This suggests a conserved role in regulating anthocyanin biosynthesis. To determine the subcellular localisation of CcMYB17, we transiently expressed a CcMYB17‐eGFP fusion protein in *Nicotiana tabacum* epidermal cells. Confocal microscopy analysis confirmed that the CcMYB17‐eGFP signal was exclusively localised to the nucleus (Supporting Information S6: Figure S6), consistent with its predicted function as a transcription factor.

To further assess whether the expression pattern of *CcMYB17* corresponds to anthocyanin accumulation at the tissue level, we performed qRT‐PCR on different organs in GT. *CcMYB17* transcript levels were predominantly detected in stems, with moderate expression in fruits, and were barely detectable in roots (Supporting Information S7: Figure S7). This tissue‐specific expression pattern closely mirrors the anthocyanin deposition in GT, where stems display the most intense red pigmentation and fruits also develop visible red coloration. These results reinforce the conclusion that *CcMYB17* is a core regulator of pigmentation in camphor.

### 2.4 CcMYB17 Directly Activates the Promoters of *CcLDOX* and *CcDFR*

**Figure 5:**
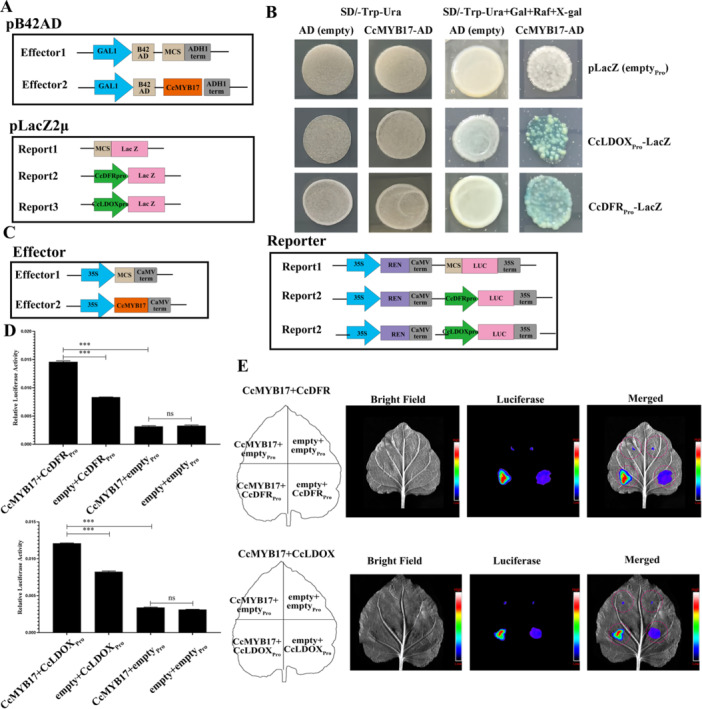
CcMYB17 directly activates the promoters of *CcLDOX* and *CcDFR*. (A and B) Yeast one‐hybrid (Y1H) assay. (A) Schematic of the Y1H bait (promoter‐pLacZ2µ) and prey (pB42AD‐CcMYB17) vectors. (B) Interaction testing on SD/‐Ura/Trp/X‐gal media. CcMYB17 binds to the promoters of *CcDFR* and *CcLDOX*, as indicated by blue coloration. (C–E) Dual‐luciferase reporter assay in *Nicotiana benthamiana* leaves. (C) Schematic of the effector (pGreenII‐62sk‐CcMYB17) and reporter (promoter‐pGreenII‐0800‐LUC) constructs. (D) Quantitative measurement of relative LUC/REN activity. CcMYB17 significantly activates the promoters of *CcDFR* and *CcLDOX*. Data represent mean ± SD from three different sets of experiments. Statistical significance was analysed using one‐way ANOVA. Asterisks indicate a significant difference (***p < 0.001), and ns indicate no significance. (E) in vivo luminescence imaging corroborates the quantitative results, showing a strong LUC signal only when CcMYB17 is co‐infiltrated with the *CcDFR* or *CcLDOX* promoters. [Color figure can be viewed at wileyonlinelibrary.com]

Previous studies have identified that MYB17‐LDOX/DFR is a key module in anthocyanin synthesis (Liu et al. 2022; Wang et al. 2022; Wu et al. 2025). Yeast one‐hybrid (Y1H) and dual‐luciferase reporter assays were performed to investigate whether CcMYB17 directly regulates *LDOX* and *DFR* in camphor trees. The Y1H results demonstrated that CcMYB17 binds directly to the promoters of *CcDFR* and *CcLDOX*, as indicated by the significant blue coloration (Figure 5A,B). Consistent with this, the dual luciferase assay showed that CcMYB17 significantly activated the promoters of *CcDFR* and *CcLDOX* (Figure 5C–E). Collectively, these findings suggested that CcMYB17 promoted anthocyanin accumulation by directly binding to and activating the promoters of two key biosynthetic genes, *CcDFR* and *CcLDOX*.

### 2.5 Functional Characterisation of *CcMYB17 in Planta*

**Figure 6:**
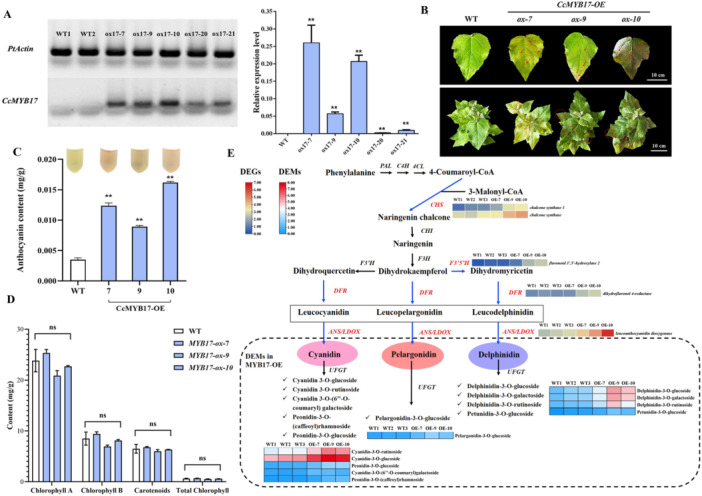
Functional characterisation of *CcMYB17* in transgenic poplar. (A) Semi‐quantitative RT‐PCR and qRT‐PCR analysis of *CcMYB17‐*overexpressing (OX) poplar lines. (Top) Semi‐quantitative RT‐PCR confirms the presence of the transgene. (Bottom) qRT‐PCR analysis of *CcMYB17* expression levels in wild‐type (WT) and five independent OX lines. Lines *OX‐7*, *OX‐9* and *OX‐10*, showing the highest expression, were selected for further analysis. The data represent the mean ± SD from three biologically independent experiments. The asterisks represent significance, where two asterisks indicates *p* < 0.01; (B) Phenotype of 2‐month‐old wild‐type (WT) and selected *CcMYB17*‐OX poplar lines (*OX‐7*, *OX‐9*, *OX‐10*). Overexpression of *CcMYB17* leads to dark leaf coloration with distinct red pigmentation (indicated by arrows). (C) Total anthocyanin content in leaves from 2‐month‐old WT and *CcMYB17‐*OX lines. Anthocyanin levels are significantly elevated in the overexpression lines. Data represent mean ± SD from three different sets of experiments. Statistical significance was analyzed using one‐way ANOVA. Asterisks indicate a significant difference (**p < 0.01) compared to WT. (D) Chlorophyll content in wild‐type (WT) and *CcMYB17*‐overexpressing (OX) poplar lines. The data represent the mean ± SD from three biologically independent experiments. (E) Expression profiles of key structural genes in the anthocyanin biosynthesis pathway in WT and *CcMYB17*‐overexpressing (OX) poplar lines. The heatmap displays log_2_‐transformed FPKM values of differentially expressed genes (DEGs). [Color figure can be viewed at wileyonlinelibrary.com]

To further determine the specific role of *CcMYB17* in anthocyanin synthesis, and due to the difficulty of generating transgenic camphor trees, we utilised a poplar model system to characterise the function of *CcMYB17* in anthocyanin accumulation *in planta*. We generated five independent stable *CcMYB17*‐overexpressing (OX) transgenic poplar lines (Supporting Information S8: Figure S8). Semi‐quantitative RT‐PCR and qRT‐PCR analysis confirmed transgene expression, with lines *OX‐7*, *OX‐9* and *OX‐10* showing the highest expression levels (Figure 6A) and were, therefore, selected for further phenotypic characterisation. At the 2‐month growth stage, the leaves of the three selected OX lines (*OX‐7*, *OX‐9* and *OX‐10*) exhibited a dark coloration with distinct red spots, in contrast to the green leaves of the wild type plants (Figure 6B). Notably, the pigmentation was not uniformly distributed across the leaf surface but appeared as distinct red spots. Consistent with this visual phenotype, the total anthocyanin content in the OX lines was significantly higher than in the wild types (Figure 6C). These results demonstrate that *CcMYB17* promotes anthocyanin synthesis *in planta*. To determine whether alterations in chlorophyll or carotenoid levels also contributed to leaf coloration, we measured chlorophyll a, chlorophyll b and carotenoid contents in WT and the three *CcMYB17*‑OX lines. No significant differences were observed between WT and OX lines for any of these pigments (Figure 6D). This finding indicates that the colour change in *CcMYB17*‐overexpressing poplar leaves is primarily driven by increased anthocyanin accumulation, rather than by chlorophyll degradation.

### 2.6 CcMYB17 Regulating Specific Anthocyanin Accumulation in the Transgenic Poplar

To characterise the *CcMYB17* transcriptional activities and effect on anthocyanins biosynthesis *in planta*. We performed RNA‐seq and targeted metabolomic analysis on leaves from wild‐type (WT) and three overexpression lines (*OX‐7*, *OX‐9*, *OX‐10*). Targeted metabolomics identified 11 anthocyanin‐related metabolites that were significantly upregulated in the *CcMYB17*‐OX lines compared to WT (Figure 6E). Among these, Cyanidin‐3‐O‐glucoside and Cyanidin‐3‐O‐rutinoside are the most abundant cyanidin derivatives and showed a significant increase. We also observed a significant increase in delphinidin derivatives, including Delphinidin‐3‐O‐glucoside, Delphinidin‐3‐O‐galactoside, and Delphinidin‐3‐O‐rutinoside. The synergistic accumulation of these red cyanidin‐ and blue or purple delphinidin‐based pigments explains the purple‐red leaf coloration in the transgenic plants.

RNA‐seq analysis revealed 185 DEGs in the *CcMYB17*‐OX lines compared with WT (Supporting Information S12: Table S1). KEGG and GO enrichment analysis confirmed that these DEGs were predominantly associated with the flavonoid biosynthetic pathway (Supporting Information S9: Figure S9). Notably, key anthocyanin biosynthetic genes, including *F3’5'H, LDOX, CHS* and *DFR*, were significantly upregulated (Supporting Information S14: Table S3). To directly validate whether *F3’5'H, LDOX, CHS* and *DFR* are upregulated in the *CcMYB17*‐overexpressing poplar lines, we performed qRT‐PCR analysis on the same leaf samples used for metabolite and transcript profiling. *F3’5'H, LDOX, CHS* and *DFR* were significantly upregulated in the three OX lines (*OX‐7*, *OX‐9*, *OX‐10*) compared to WT (Supporting Information S10: Figure S10). This indicates that *CcMYB17* promotes cyanidin and delphinidin accumulation by activating the expression of these critical structural genes.

## 3 Discussion

The camphor tree is a cornerstone species for urban greening in southern China, yet its landscaping values is limited by a lack of colour variation. The novel ornamental variety ‘Gantong 1’ (GT) with its distinctive stem coloration, represents a significant advancement for landscape application. Unlike most commercial varieties, GT exhibited dynamic colour changes across seasons. However, the molecular mechanism underlying this trait has not been explored. Our elucidation of the pathways and regulators governing the pigment accumulation in GT provides crucial insights for the molecular breeding of ornamental traits in camphor trees.

Plant coloration is determined by pigments such as chlorophyll, carotenoids, and anthocyanins (Ghosh et al. 2022). In GT, the mature leaves transition to yellow‐green in early summer, correlating with increased chlorophyll and decreased anthocyanin levels, demonstrating how the interplay of these pigment shapes leaf colour. More critically for the unique stem phenotypes, we found that chlorophyll and carotenoid levels in GT stem were significantly lower than in CK, while anthocyanin content was substantially higher (Figure 1). This indicates that anthocyanin accumulation combined with chlorophyll degradation is the primary factor responsible for the red stem coloration in GT.

Anthocyanins, a class of flavonoid, confer orange‐red to blue‐purple hue in plants (Han et al. 2010). The content and types of anthocyanins vary with tree species. For example, the main anthocyanin in apple and pear is cyanidin‐3‐O‐galactoside (Clayton‐Cuch et al. 2023; Du et al. 2025), while in blood‐fleshed peaches, cyanidin‐3‐glucoside is the main anthocyanin (Song et al. 2025). Our integrated transcriptomic and metabolomic analysis revealed that the red stem of GT is associated with substantial accumulation of specific anthocyanins, including cyanidin, pelargonidin, and peonidin. Cyanidin‐3‐O‐glucoside was identified as the most abundant anthocyanin and the key pigment in camphor tree stem. In CK trees, the brown stem results from cyanidin combined with background levels of chlorophyll and carotenoids. In GT, the significant reduction of these masking pigments, coupled with cyanidin accumulation, leads to the dark red stem. The results of PCA analysis showed that Cyanidin‐3‐O‐glucoside, Cyanidin‐3‐O‐galactoside, Cyanidin‐3‐O‐rutinoside, Pelargonidin‐3‐O‐(6‐O‐malonyl‐beta‐d‐glucoside), Cyanidin‐3,5‐O‐diglucoside are of great importance in influencing GT coloration (Figure 3). Transcriptional evidence suggested that 26 genes in phenylpropanoid and flavonoid biosynthesis pathway were associated with 13 flavonoid metabolites, confirming that the coloration is driven by the altered expression of anthocyanin structural genes.

The MYB‐bHLH‐WD40 (MBW) complex is a master regulator of anthocyanin biosynthesis, with R2R3‐MYB transcription factors as essential players (Wang et al. 2020; Luan et al. 2022; Zhang et al. 2025). In Arabidopsis, *AtMYB75* (*PAP1*), *AtMYB90* (*PAP2*), *AtMYB113* and activate genes like *CHS, DFR, LDOX* to induce anthocyanin accumulation (Stracke et al. 2001; Zimmermann et al. 2004; Gonzalez et al. 2008). PdMYB113 was identified as a key regulator regulating the red coloration of *Populus deltoides* by controlling the expression of *CHS* and *LDOX* (Li et al. 2025). Our phylogenetic and correlation analysis identified CcMYB17, homologous to AtMYB113, as a key regulator in this pathway in camphor trees. The functional characterisation of *CcMYB17* in poplar validated its binding to *CcDFR* and *CcLDOX* promoters, promoting anthocyanin accumulation, resulting in darker leaves with prominent red pigmentation (Figure 6). An intriguing observation in our study is that *CcMYB17*‐overexpressing poplar lines exhibited only partial and spotted pigmentation on leaves, rather than the uniform red coloration seen in GT stems. Several non‐mutually exclusive explanations may account for this difference. First, species‐specific differences in the availability of interacting partners or substrate preferences of the anthocyanin pathway between poplar and camphor could affect the activity of heterologous *CcMYB17*. In Arabidopsis, the MBW complex requires specific combinations of MYB, bHLH, and WD40 components for full activity, and endogenous bHLH partners in poplar may not fully substitute for camphor‐specific co‐factors. Second, anthocyanin biosynthesis is known to be under distinct developmental and environmental control in different organs. The leaf epidermis may have a higher threshold for pigment induction or different metabolic fluxes compared to the stem periderm. Third, post‐transcriptional or post‐translational modifications of CcMYB17 in poplar may modulate its transactivation activity, stability, or subcellular localisation. Fourth, the endogenous anthocyanin regulatory network in poplar may compete with or modify the function of the introduced CcMYB17. Poplar contains its own MYB activators, and heterologous CcMYB17 might be integrated into existing complexes with varying efficiency, leading to stochastic activation in only a subset of cells. Finally, we found that chlorophyll a, chlorophyll b, and carotenoid levels did not differ significantly between wild‐type and *CcMYB17*‐OX poplar leaves (Figure 6D), in sharp contrast to the GT stem phenotype where reduced chlorophyll and carotenoids accompanied anthocyanin elevation (Figure 1C). The persistence of background green pigmentation from chlorophyll in transgenic leaves may mask anthocyanin‐derived purple‐red hues in many cells, further contributing to the spotted coloration. This pattern of partial pigmentation is not uncommon in transgenic plants overexpressing anthocyanin regulators from other species and likely reflects a threshold effect where only cells reaching a critical level of regulatory complex activity accumulate visible pigment.

Beyond the downstream targets of *CcMYB17*, an equally important question is what drives its elevated expression in GT in the first place. To test whether cis‐regulatory variation in the CcMYB17 promoter is responsible, we cloned and compared the approximately 2‐kb promoter sequences from GT and CK (Supporting Information S11: Figure S11). The two promoters were highly similar, with no large insertions or deletions. However, PlantCARE analysis revealed three specific differences in known cis‐acting elements. Compared with CK, the GT promoter contains one fewer STRE (stress response element) and one fewer as‐1 (activation sequence‐1) motif, but one additional CGTCA‐motif (MeJA‐responsiveness element). STRE mediates transcriptional responses to various abiotic stresses, as‐1 is involved in auxin and salicylic acid signalling, and the CGTCA‐motif is associated with methyl jasmonate induction (Supporting Information S18: Table S7). Although these variations could subtly modulate the responsiveness of the *CcMYB17* promoter to hormonal or environmental signals, their limited number and close identity of the two promoter sequences suggest that cis‐regulatory mutations are unlikely to be the primary cause of the constitutive high level *CcMYB17* expression observed in GT stems across multiple seasons. A more plausible explanation is that differences in upstream transcription factors or epigenetic modifications drive its upregulation. Identifying these upstream regulators will be essential to fully elucidate the regulatory cascade underlying GT stem coloration.

**Figure 7:**
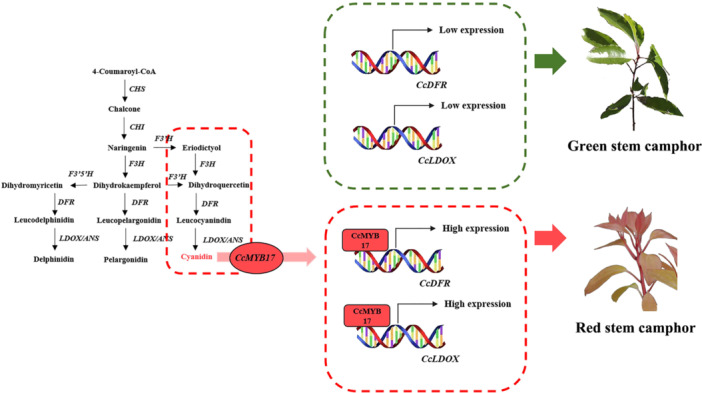
The model of *CcMYB17* regulating anthocyanin accumulation in *C. camphora*. [Color figure can be viewed at wileyonlinelibrary.com]

The specific hue of anthocyanin‐pigmented tissues depends on the type of anthocyanin accumulated; cyanidin and peonidin produce red tone, while delphinidin and petunidin contribute to the blue to purple‐red shift. In *CcMYB17*‐overexpressing poplar, we observed a significant increase in both cyanidin derivatives (e.g., Cyanidin‐3‐O‐glucoside and Cyanidin‐3‐O‐rutinoside) and delphinidin derivatives (e.g., Delphinidin‐3‐O‐glucoside, Delphinidin‐3‐O‐galactoside, and Delphinidin‐3‐O‐rutinoside). The synergistic accumulation of these red and blue pigments explains the purple‐red coloration of the transgenic leaves. This specific metabolite profile is directed by the transcriptome. We found that the structural genes *DFR*, and *LDOX* were significantly upregulated in transgenic poplar trees. In summary, the concerted activation of this biosynthetic network by CcMYB17‐DFR/LDOX is thus the primary cause of the purple‐red pigmentation in the transgenic lines (Figure 7), which provides genetic resources for breeding new varieties of woody plants.

## 4 Materials and Methods

### 4.1 Plant Materials and Sampling

In the experiment, the GT mutant plants were propagated via cuttings, and CKs were the half‐sib progeny seedlings derived from the same mother tree of the GT mutant. All trees were planted at the Jiangxi Academy of Sciences (28°69′N, 116°00′E) under identical growth conditions and management practices. All experiments were performed with three independent biological replicates, and each biological replicate consisted of samples from three individual trees. The sampling time was divided into six time points (January, April, May, July, August, and December) for pigment extraction, transcriptomic and metabolomic analysis.

For anthocyanins content and gene expression analysis at 2‐month stage under the control growth room conditions, the WT, *OX‐7*, *OX‐9* and *OX‐10* plants were grown in pots under 16‐h light/8‐h dark photoperiod conditions at 26°C.

### 4.2 Determination of Plant Pigment

The RHS plant colour card of the Royal Garden Society of England was used for colour comparison throughout the year, and the stem colour were recorded and photographed.

The contents of chlorophyll and carotenoids were determined by dimethyl sulfoxide (DMSO) extraction method. Weigh 0.1 g of fresh stem and put it in a 10 mL centrifuge tube, add 5 mL of DMSO, and extract them at room temperature and dark for 24 h until the colour of stem completely fades to transparent shape. A total of 200 μL of supernatant was taken and the absorbance values at 470, 649 and 665 nm were measured by enzyme‐linked immunosorbent assay.

$$\begin{array}{l} \text{Chla}\;\left(\text{mg}/g\right)=\left(13.95\times \text{A665}-6.88\times \text{A649}\right)\times \text{extract}\;\text{volume}\;\left(\text{mL}\right)\\ \times \;\text{dilution ratio}/\left(\text{sample}\;\text{fresh}\;\text{weight}\;\left(g\right)\times 1000\right), \end{array}$$

$$\begin{array}{l} \text{Chlb}\;\left(\text{mg}/g\right)=\left(24.95\times \text{A649}-7.32\times \text{A665}\right)\times \text{extract}\;\text{volume}\;\left(\text{mL}\right)\\ \times \;\text{dilution}\;\text{ratio}/\left(\text{sample}\;\text{fresh}\;\text{weight}\;\left(g\right)\times 1000\right), \end{array}$$

$$\begin{array}{l} \text{Chlt}\;\left(\text{mg}/g\right)=\left(6.63\times A665+18.07\times A649\right)\times \text{extract}\;\text{volume}\;\left(\text{mL}\right)\\ \times \;\text{dilution}\;\text{ratio}/\left(\text{sample}\;\text{fresh}\;\text{weight}\;\left(g\right)\times 1000\right), \end{array}$$

Car(mg/g)=(1000×A470−2.05×Chla−114.8×Chlb)/245.

Anthocyanins were extracted according to Gonzalez‐montelongo method. Weigh 0.1 g of sample into 15 mL centrifuge tubes, add 10 mL of 0.1 mol/L hydrochloric acid ethanol solution to each tube, and extract it in 60°C water bath for 1 h. with 0.1 mol/L hydrochloric acid ethanol solution as blank control, determine the absorbance value of sample extract at 530, 620 and 650 nm.

$${\text{OD}}_{\lambda }=\left({\text{OD}}_{530}*-{\text{OD}}_{620}\right)-0.1\left({\text{OD}}_{650}-{\text{OD}}_{620}\right).$$

Anthocyanin content (nmol/g) = OD_λ_/4.62 × 10^4^ × extract volume (mL)/sample weight (g) × 1 000 000.

### 4.3 Metabolomic Profiling Analysis

Anthocyanin‐targeted metabolomic analysis was performed by Metware Biotechnology Co. Ltd. (Wuhan, Hubei, China). 1 g of the stem sample was weighed and vacuum‐freeze‐dried in a freeze dryer. The sample was ground into powder for UPLC‐MS/MS analysis. Data acquisition was carried out by ultra performance liquid chromatography (UPLC) and Tandem Mass Spectrometry (MS/MS). After processing the mass spectrometry data, based on the self‐built metware metabolism database MWDB, the metabolites were qualitatively and quantitatively analyzed by mass spectrometry. The differential metabolites were screened using VIP ≥ 1, difference multiple value ≥ 2 or ≤ 0.5 as the screening criteria. For downstream analysis, metabolites with a mean relative content > 0.5 across all samples were considered major contributors to stem coloration and selected for further analysis. This threshold was determined based on the natural distribution of metabolite abundances to focus on the most abundant anthocyanins likely to determine the visible phenotype. The complete dataset of all 46 identified anthocyanin metabolites, including their relative contents in GT and CK across all six time points, is provided in Supporting Information S13: Table S2.

### 4.4 Transcriptome Sequencing and Analysis

Quantitative samples were homogenised with TRIzol reagent (Invitrogen, USA). Transcriptome libraries were constructed using the Oligo (dT) magnetic bead enrichment method, and second‐generation transcriptome sequencing using the Illumina Novaseq. 6000 platform were mapped to the reference genomic sequences of *C. camphora* using HISAT2. Then, gene annotation was performed using National Center for Biotechnology Information (NCBI) nonredundant protein sequences (NR), EuKaryotic Orthologous Groups (KOG), Kyoto Encyclopedia of Genes and Genomes (KEGG), and Gene Ontology (GO).

### 4.5 Gene Cloning and Bioinformatics Analysis

The full‐length sequence of *CcMYB17* was amplified with two primers from the cDNA of GT. The primers used are shown in Supporting Information S12: Table S1. The amino acid sequences of MYB in each species were compared using DNAMAN. The amino acid sequences of MYB proteins from *Arabidopsis thaliana*, *Populus trichocarpa* and *Eucalyptus grandis* were retrieved from TAIR (https://www.arabidopsis.org), Phytozome (https://phytozome-next.jgi.doe.gov), or NCBI GenBank. A phylogenetic tree was constructed with IQ‐TREE 2.3.6 (Minh et al. 2020), and the proteins were grouped according to the tree topology. The best model chosen by the software was JTT + R8. The generated align.fasta.treefile was subsequently visualised using iTOL (https://itol.embl.de, Letunic and Bork 2024). Classify the members of MYB using NCBI‐Conserved Domain Data (http://www.ncbi.nlm.nih.gov/Structure/cdd/wrpsb.cgi; CDD).

### 4.6 Gene Construction

CcMYB17 was seamlessly recombined into the plant binary expression vector pBI121‐eGFP, which was double‐digested with *Xba* I and *Sma* I restriction endonucleases. Subsequently, the target fragment with sticky ends was ligated to the linearised vector via homologous recombination‐mediated ligation. the ligation products were transformed into Top 10 competent *Escherichia coli* cells. PCR‐based colony screening and sequencing using vector‐specific primers confirmed the successful construction of the recombinant expression vector pBI121‐CcMYB17‐eGFP. The primers used are shown in Supporting Information S12: Table S1.

### 4.7 Subcellular Localisation

*Agrobacterium tumefaciens* strains harbouring pBI121‐eGFP and pBI121‐CcMYB17‐GFP constructs were co‐transformed with NLS‐mCherry into tobacco leaves via the vacuum infiltration. After 3 days, the localisation of the fusion protein was observed through fluorescence image under 488 and 587 nm excitation light, respectively.

### 4.8 Plant Genetic Transformation

*A. tumefaciens*‐mediated vacuum infiltration was used for plant transformation. OD_600_ = 0.6–0.8 to resuspend the bacterial cells in MS suspension and soak 84 K poplar leaf blocks pre‐cultured for 3 days in bacterial solution for 10–15 min. Transfer the leaves to differentiation medium containing 30 mg/L Kan and 300 mg/L Cef for screening and cultivation. PCR detection was performed on the genomic DNA extracted from wild‐type and transgenic poplar. RT‐PCR were performed on wild‐type and transgenic poplar leaves grown for 1 month.

### 4.9 Anthocyanin Extraction

Wild‐type and transgenic poplar stem segments were inoculated into rooting medium and cultivate them in the same environment for 30 days. Transplant the tissue culture seedlings into the soil and cultivate them in the same environment for 1 month. Take leaves for phenotype observation, and use the Total Anthocyanin Content Kit (Grace Biotechnology Co. Ltd, China) to determine anthocyanin content.

### 4.10 Y1H

The CDS of *CcMYB17* was subcloned into the pB42AD vector to generate pB42AD‐CcMYB17 plasmid. The DNA sequence upstream of the ATG start codon of anthocyanin synthesis genes were inserted into the pLacZi2µ vector. Strain EGY48 was used for conversion. The transformed yeast cells were inoculated into (SD/−Trp/‐Ura) medium with X‐Gal. The primers used are shown in Supporting Information S12: Table S1.

### 4.11 Dual‐Luciferase Assay

The CDS sequence of *CcMYB17* transcription factor was constructed by homologous recombination method into pGreenII‐62‐sk vector driven by 35S promoter for expression of effector protein. The promoter sequences of *CcDFR* and *CcLDOX* were constructed into pGreenII‐0800. pGreenII‐62sk‐empty and pGreenII‐62sk‐CcMYB17 infection solutions were mixed with pGreenII‐0800‐empty, pGreenII‐0800‐CcDFRpro and pGreenII‐0800‐CcLDOXpro by volume 1:1, respectively, and vacuum injected on different parts of the same tobacco leaf. The back of tobacco leaves was smeared with fluorescein potassium salt working solution and left for 5 min in the dark. The intensity of fluorescence signal in leaves coated with fluorescein substrate d‐luciferin was detected by in vivo luminescence imaging system (Tannon 4600SF), and photographed and recorded. Add 1× cell lysis buffer lysate to tobacco leaf discs of the same weight, and take the supernatant to the cell culture plate for detection, with three biological replicates in each group. Luciferase substrate detection reagent was added to the supernatant and quickly mixed. The activity of firefly luciferase reporter gene was detected by the microplate reader. Renilla substrate working solution was added to detect the activity of Renilla luciferase reporter gene.

### 4.12 qRT‐PCR

The stems of GT and CK in different months, different tissues (roots, stems, leaves, flowers, fruits) and leaves of wild‐type and transgenic poplars grown for 1 month were detected by qRT PCR. *PtrActin7* and *CcActin* were used as internal reference genes to detect changes in the expression level of *CcMYB17* gene in the plants, and the expression of *F3’5'H, LDOX, CHS* and *DFR* in OX lines were also examined. Primers are listed in Supporting Information S12: Table S1.

### 4.13 Statistics

SPSS statistics 18 software was used for data processing, one‐way analysis of variance, Duncan multiple comparison and Pearson correlation analysis. All charts were drawn by Graphpad prism 8 and origin 2019 software. WGCNA analysis was conducted using Metware cloud platform (https://cloud.metware.cn/). Intramodular total connectivity (ktotal) for each gene was calculated as the sum of absolute Pearson correlation coefficients between that gene and all other genes within the same module. This metric quantifies the centrality of a gene within its co‐expression network. The principal component analysis (PCA) was conducted using the prcomp function in R (Version 4.4.1). The results of the PCA were visualised using the ggplot2 package.

## Conflicts of Interest

The authors declare no conflicts of interest.

## Supporting information


**Figure S1:** KEGG analysis of differentially expressed genes of GT and CK in different months.

**Figure S2:** Expression profiles of key structural genes in the anthocyanin biosynthesis pathway in GT and CK trees across seasons. The heatmap displays log2‐transformed FPKM values of differentially expressed genes (DEGs) for each month.

**Figure S3:** qRT‑PCR validation of *CcMYB17* expression in GT and CK trees across seasons.

**Figure S4:** Phylogenetic tree analysis of CcMYB17 and MYB family in *Arabidopsis thaliana, Populus trichocarpa* and *Eucalyptus grandis*.

**Figure S5:** Domain analysis of CcMYB17.

**Figure S6:** Subcellular localization of a CcMYB17‐eGFP fusion protein in *Nicotiana tabacum* epidermal cells. The eGFP signal (green) is exclusively co‐localized with Nuclear‐Localization‐Signal (NLS)‐mCherry fusion protein to the nucleus (red). Bright field and merged images are shown. Scale bar=20 µm.

**Figure S7:** qRT‑PCR validation of *CcMYB17* expression in different tissues in GT.

**Figure S8:** DNA detection of *CcMYB17*‑overexpressing poplar.

**Figure S9:** Transcriptome analysis of *CcMYB17*‑overexpressing poplar and WT. (A) KEGG analysis of differentially expressed genes of *CcMYB17* transgenic poplar and WT; (B) Volcano plot of differentially expressed genes of *CcMYB17* transgenic poplar and WT.

**Figure S10:** qRT‑PCR validation of *F3’5'H, LDOX, CHS*, and *DFR* expression in WT and *CcMYB17*‑overexpressing poplar lines.

**Figure S11:** Comparison the *CcMYB17* promoter sequences between GT and CK.

**Table S1:** Primer sequence.

**Table S2:** Anthocyanin metabolites in GT and CK stems.

**Table S3:** Differential gene expression between *CcMYB17*‐OX and WT.

**Table S4:** Ktotal of all genes in three modules.

**Table S5:** Correlation between *CcMYB17* and anthocyanin structural genes in three modules.

**Table S6:** Correlation between metabolites and anthocyanin structural genes in three modules.

**Table S7:** Comparison the cis ‐ acting elements of *CcMYB17* promoters between GT and CK by PlantCARE.

## Data Availability

The data that supports the findings of this study are available in the Supporting Information of this article.
