# Supplementary material for: MYB17‐DFR/LDOX Module Positively Regulates Cyanidin Deposition in Cinnamomum Camphora
Source: Plant Cell Environ. 2026 Jun 12;49(9):6873–85. doi: 10.1111/pce.70664 (PMC13436556; doi:10.1111/pce.70664)
Supplement: Supplementary file 1 [file PCE-49-6873-s014.pdf]

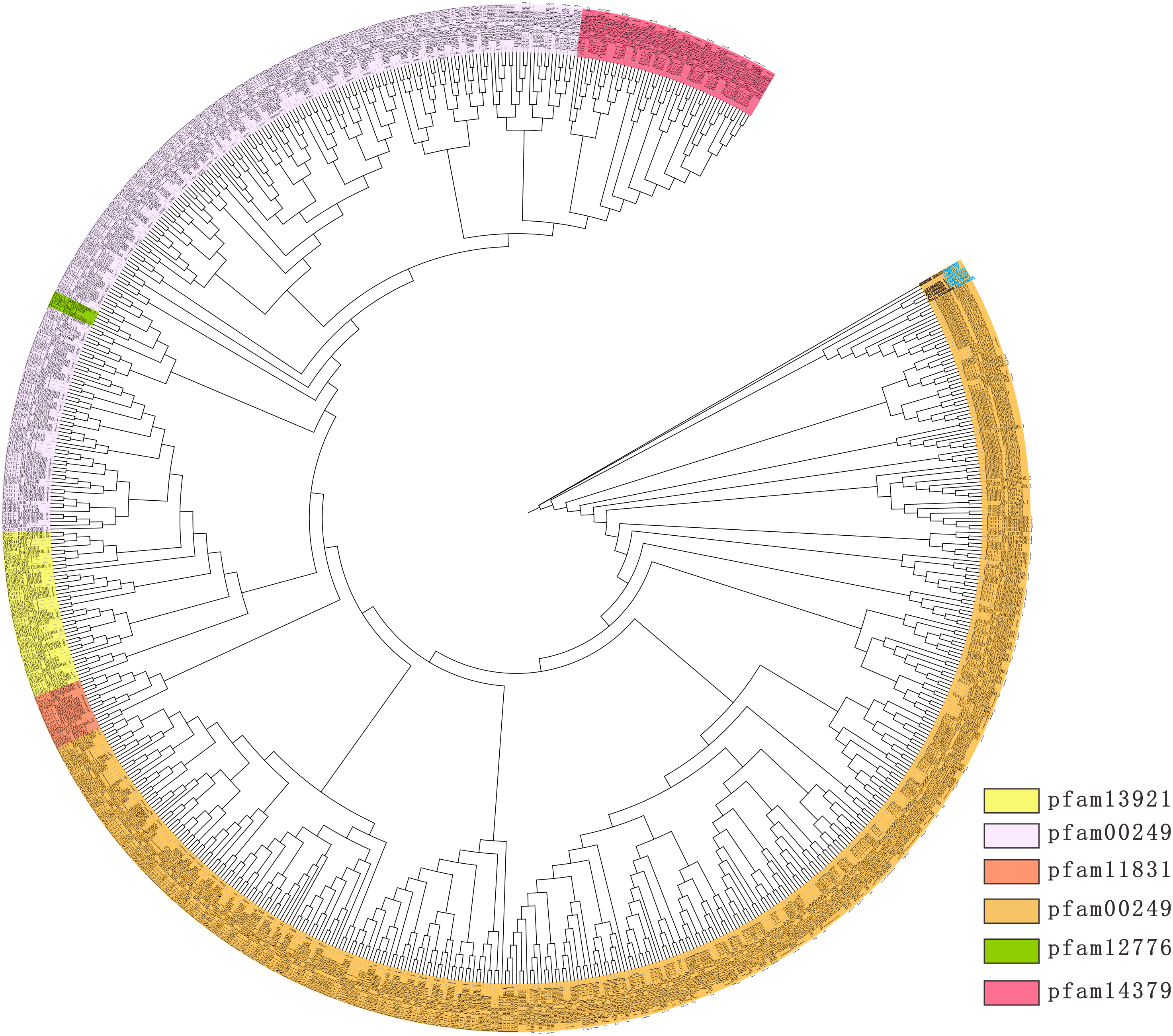

pfam13921
pfam00249
pfam11831
pfam00249
pfam12776
pfam14379
